# Novel MRI tests of orocecal transit time and whole gut transit time: studies in normal subjects

**DOI:** 10.1111/nmo.12249

**Published:** 2013-10-25

**Authors:** G Chaddock, C Lam, C L Hoad, C Costigan, E F Cox, E Placidi, I Thexton, J Wright, P E Blackshaw, A C Perkins, L Marciani, P A Gowland, R C Spiller

**Affiliations:** * Sir Peter Mansfield Magnetic Resonance Centre, School of Physics and Astronomy, University of NottinghamNottingham, UK; † Nottingham Digestive Diseases Centre, School of Medicine, University of NottinghamNottingham, UK; ‡ Nottingham Digestive Diseases Biomedical Research Unit, Nottingham University Hospitals, University of NottinghamNottingham, UK; § GI Surgery, Nottingham University HospitalsNottingham, UK; ¶ Medical Physics & Clinical Engineering, Nottingham University HospitalsNottingham, UK; ** Radiological & Imaging Sciences, School of Medicine, University of NottinghamNottingham, UK

**Keywords:** correlation, marker capsule, MRI, transit time

## Abstract

**Background:**

Colonic transit tests are used to manage patients with Functional Gastrointestinal Disorders. Some tests used expose patients to ionizing radiation. The aim of this study was to compare novel magnetic resonance imaging (MRI) tests for measuring orocecal transit time (OCTT) and whole gut transit time (WGT), which also provide data on colonic volumes.

**Methods:**

21 healthy volunteers participated. Study 1: OCTT was determined from the arrival of the head of a meal into the cecum using MRI and the Lactose Ureide breath test (LUBT), performed concurrently. Study 2: WGT was assessed using novel MRI marker capsules and radio-opaque markers (ROMs), taken on the same morning. Studies were repeated 1 week later.

**Key Results:**

OCTT measured using MRI and LUBT was 225 min (IQR 180–270) and 225 min (IQR 165–278), respectively, correlation r_s_ = 0.28 (ns). WGT measured using MRI marker capsules and ROMs was 28 h (IQR 4–50) and 31 h ± 3 (SEM), respectively, correlation r_s_ = 0.85 (p < 0.0001). Repeatability assessed using the intraclass correlation coefficient (ICC) was 0.45 (p = 0.017) and 0.35 (p = 0.058) for MRI and LUBT OCTT tests. Better repeatability was observed for the WGT tests, ICC being 0.61 for the MRI marker capsules (p = 0.001) and 0.69 for the ROM method (p < 0.001) respectively.

**Conclusions & Inferences:**

The MRI WGT method is simple, convenient, does not use X-ray and compares well with the widely used ROM method. Both OCTT measurements showed modest reproducibility and the MRI method showed modest inter-observer agreement.

**Key Messages**

1. Colonic transit tests are often useful in managing functional gastrointestinal disorders (FGIDs).
2. We used serial MRI to assess orocecal transit (OCTT) of the head of a test meal and the position of water filled capsules taken 24 hours before as markers of whole gut transit (WGT).
3. The new MRI techniques were compared with the Lactose Ureide breath test to measure OCTT and the Radioopaque Marker (ROM) method to measure the WGT.
4. The MRI marker capsule technique compared favorably with the standard ROM method for measuring WGT and being non-invasive and non-ionizing, has significant advantages in patients with FGIDs, many of whom are young females.

## Introduction

Functional gastrointestinal disorders (FGIDs) account for ~40% of all gastrointestinal secondary care referrals, so efficient diagnosis is important.^1^ The commonest diagnoses are functional constipation, functional diarrhea, and irritable bowel syndrome.^2^ Currently, diagnosis is based on reported symptoms, which can sometimes be unreliable, particularly when assessing reported constipation.

Objective measurements of transit are currently the best validated biomarkers to guide treatment^3^ and predict drug effectiveness.^4^ There are a variety of tests which have been developed to measure the orocecal transit time (OCTT) with gamma scintigraphy, imaging the small bowel transit of a tracer, considered as the gold standard.^3^ This method is limited in use as it is costly, poorly standardized, and exposes the patient to ionizing radiation which would limit its repeated use. In our study, we used two alternative methods to measure OCTT, the Lactose Ureide breath test (LUBT) and magnetic resonance imaging (MRI), both of which have the advantage of not using ionizing radiation. There are also a number of techniques used to measure whole gut transit time (WGT), which is dominated by the time to transit the colon. These include radio-opaque markers (ROMs),^5^ scintigraphy,^6^ magnetic marker monitoring,^7^ wireless motility capsule,^8^ and fluorine-19 labeled MRI markers.^9^ While each method has its unique advantages most have not become widely adopted in clinical practice due to their own particular limitations.^10^ The Metcalf ROM method is probably most widely used in hospitals to assess WGT. Although it is simple and inexpensive, given that a substantial proportion of patients in whom such tests are indicated are females of child-bearing age, a major drawback of the ROM method is that it exposes to ionizing radiation. Magnetic resonance imaging can overcome some of these limitations and potentially offer tests which could be widely adopted and benefit from being non-invasive and avoid ionizing radiation.

The aim of this study was to validate two novel MRI based methods, one for measuring OCTT and one for measuring WGT and also to assess their reproducibility. We also took advantage of the extra capabilities of MRI to examine the relation between bowel habit, colonic volumes, and transit time in healthy volunteers.

## Materials and Methods

The subjects participated in two separate test–retest open label studies. Study 1 to compare an OCTT MRI method against the LUBT and Study 2 to compare a WGT MRI method against the widely used Metcalf ROM method. This protocol was registered with ClinicalTrials.gov identifier: NCT01534507. The studies were approved by the National Research Ethics Service (REC number 11/EM/0245); all volunteers gave written informed consent. The studies were carried out according to Good Clinical Practice principles.

### Subjects

Twenty-one healthy subjects (12 males, 9 females; 21–70 years) were enrolled and took part in both studies. Subjects with any previous history of gastrointestinal disease, or taking any medication known to alter bowel motility were excluded from the study. All subjects completed a MRI safety questionnaire, to exclude persons with contraindications to MRI, and a hospital and anxiety scale questionnaire. All of the 21 volunteers completed Study 1, which was repeated after a 1 week washout period, to assess reproducibility of the tests. 20 of the same 21 volunteers completed Study 2, also repeated after a 1 week washout period, to assess reproducibility.

## Study 1: orocecal transit time (OCTT)

### MRI OCTT test

Subjects attended at 08:00 am after an overnight fast and underwent a baseline MRI scan before being fed a mixed solid/liquid test meal as used in previous studies.^11^ This consisted of: 220 g creamed rice pudding (J Sainsbury plc, London, UK), 34 g seedless strawberry jam (J Sainsbury plc), uniformly mixed with 15 g course wheat bran (Holland and Barrett, Hinkley, UK), and a glass of 100 mL orange juice from concentrate (J Sainsbury plc) providing a total of 362 Kcal. Subjects were scanned every 45 min for a total of 8.5 h. They were fed a second 1000 Kcal meal at 6.5 h which consisted of: 400 g microwaveable macaroni cheese ready meal (J Sainsbury plc), 100 g strawberry cheesecake (J Sainsbury plc) and 250 mL bottled still water (J Sainsbury plc). Magnetic resonance imaging scanning was carried out on a 1.5 T Philips Achieva scanner, using a 16 channel XL torso coil. Subjects were scanned in a supine position and in between scans volunteers sat in an upright position in the waiting room. The arrival of the head of the meal into the cecum was determined from images acquired using a dual-echo 2D multi-slice FFE sequence (Echo Time 1 [TE_1_] = 4.6 ms; Echo Time 2 [TE_2_] = 6.9 ms; Repetition Time [TR] = 212 ms, Flip Angle [FA] = 80°). 24 coronal images were acquired to cover the abdomen with an acquired voxel size of 2.01 × 2.87 × 7.00 mm^3^ (reconstructed voxel size of 1.76 × 1.77 × 7.00 mm^3^), a field of view [FOV] of 450 × 360 mm^2^, and a slice thickness of 7 mm with no gaps (SENSE factor = 1.7). Images were acquired during a breath hold of 17 s. An additional single shot turbo spin echo (TSE) sequence was acquired to measure small bowel water content (SBWC)^12^ which meant subjects spent ~10 min inside the magnet for each time point.

The arrival of the head of the rice pudding meal was assessed visually using the 2D FFE images. We estimated the OCTT as the time from the firsts scan to show entry of bolus of high intensity material into the ascending colon which prior to this event had mostly a low intensity on the images. We also used the 2D FFE images to measure colonic volumes before (*t* = 360) and after (*t* = 405) the high calorie meal, as it has been implied before that clearance of the ascending colon may correlate with overall transit time.^13^ We measured colonic volumes at these particular time points after the standard meals, rather than fasting, to minimize differences which might relate to activity and feeding patterns the day before. Although our standard practice is to exclude certain foods likely to alter transit this still leaves room for lots of difference which might alter fasting volumes. Colonic volumes were measured using the software Analyze© 9.0 (Biomedical Imaging Resource, Mayo clinic, Rochester, MN, USA).

## Lactose Ureide breath test

**Figure 1:**
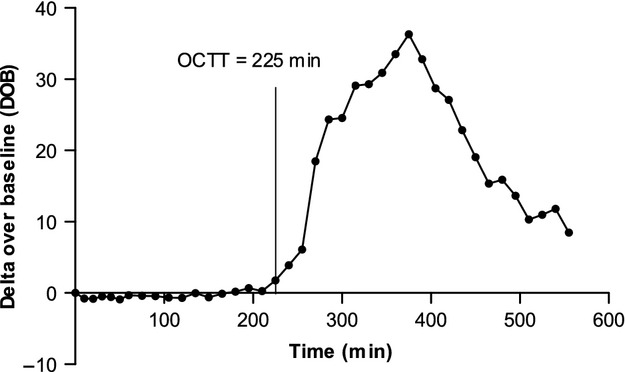
^13^C breath excretion curve. Example ^13^C breath excretion curve from Lactose Ureide Breath Test in one healthy volunteer with indication of the orocecal transit time (OCTT). The OCTT is taken as the time at which there is an increase in breath ^13^C which is 2.5 times the SD of all previous points above the running average of all previous points.

We used a previously validated LUBT protocol.^14^ The day before the test day subjects ingested 1 g (6 mmol) of unlabeled Lactose Ureide (Euriso-top®, Saint-Aubin Cedex, France) three times a day with meals (morning, afternoon, and evening), to stimulate bacterial enzyme activity to cleave the Lactose Ureide in the colon. On the test day the LUBT was performed alongside the MRI OCTT test (described above) and subjects provided a baseline breath sample before being fed a standard test meal (details above). The test meal was mixed with 500 mg ^13^C labeled Lactose Ureide (Euriso-top®, Saint-Aubin Cedex, France). Breath samples were taken every 15 min for 1 h and then every 10 min for a further 9 h. A second high calorie meal was fed 6.5 h after the test meal (details above). Breath samples were analyzed using an IRIS®-Lab analyzer machine (Wagner Analysen Technik, Bremen, Germany). Results are expressed as delta over baseline, which is the difference between the ratio of ^13^CO_2_/^12^CO_2_ in the post dose breath sample and the corresponding ratio in the baseline sample. The OCTT was taken as the time post ingestion that the increase in breath ^13^C reached 2.5 times the SD of all previous above the running average of all previous points (Fig. 1), as defined in the LUBT validation study.^14^ The OCTT was automatically determined from the breath data using an in-house program written in Matlab (MathWorks, Natick, MA, USA). The dose we used 6 mmol would not be expected to exert a significant osmotic effect and hence unlike lactulose, often used as a marker of orocecal transit, would not be expected to alter transit.

## Study 2: whole gut transit time

### MRI WGT test

**Figure 2:**
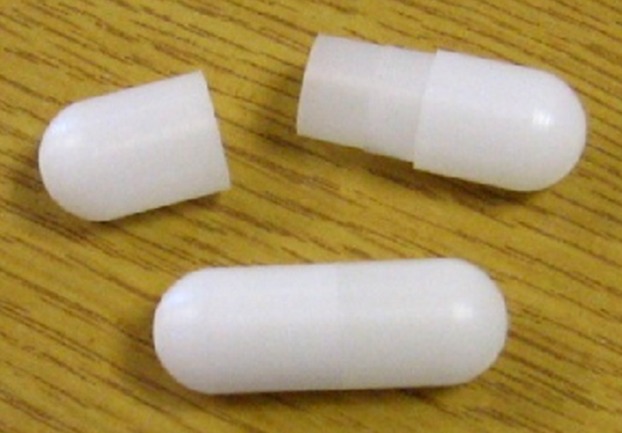
Magnetic resonance imaging (MRI) marker capsule. Example of a capsule which consists of two polyoxymethylene half shells glued together, and hand filled with 0.4 mL 15 *μ*M Gadoteric acid (Gd-DOTA). Leakage tests performed on 20% of each batch using a spectrophotometer. Capsules have the dimensions of 20 × 7 mm.

Subjects swallowed five MRI marker capsules (20 × 7 mm) at 09:00 am, 24 h before undergoing an MRI scan. The MRI marker capsules were all manufactured in-house using the biologically inert, Polyoxymethylene (Fig. 2). The capsules consist of two half-shells which were glued together using cyanoacrylate superglue. A small hole had been drilled in the top of one half-shell, so capsules could be hand filled with 0.4 mL 15 *μ*M Gadoteric acid (Gd-DOTA). A small plastic screw was then inserted into the hole, and glued with cyanoacrylate superglue to prevent leakage. Spectrophotometry was used to asses leakage on 20% of each batch of pills produced.

The MRI markers capsules were filled using the non-toxic and biocompatible Gd-DOTA, which is a complex of Gd^3+^ and the chelating agent DOTA. It is routinely used in clinical practice as an MRI imaging contrast agent, and its safety is well-documented. The agent shortens the T1 relaxation times of protons it has access to increasing the signal on T1 weighted images. Preliminary work at our centre has shown that the optimal concentration of Gd-DOTA to be used in the capsules was 15 *μ*M, which ensured that we obtain the maximum signal intensity from the capsules on our T1 weighted images.^15^ This concentration was achieved by diluting 1 mL of Gd-DOTA, at a concentration of 280 mg/mL, with 33 mL of distilled water.

**Figure 3:**
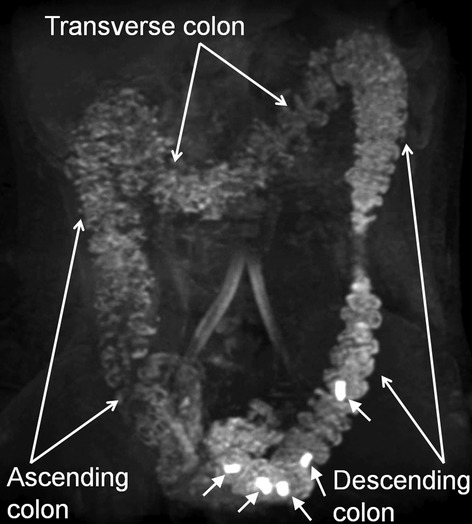
Maximum intensity projection (MIP) magnetic resonance imaging (MRI) image. Showing five MRI marker capsules in the colon (indicated by close arrows). Images created using water only images acquired using the T1 weighted multi-echo FFE pulse sequence. Such images were used to locate the number and position of capsules remaining at 24 h to calculate a whole gut transit time.

Subjects were scanned in a 3 T Philips Achieva MRI scanner using a multi-transmit body coil. Coronal scans were obtained at two stations with a 30 mm overlap using two different sequences. Firstly, a T1 weighted 3D TFE sequence (TE = 1.3 ms; TR = 2.9 ms, FA = 10°, FOV = 250 × 398 × 160 mm^3^, Acquired resolution [AQR] = 2.3 × 2.3 × 4 mm^3^), was used to count and locate the number of capsules remaining in the colon at 24 h. Secondly, a multi-echo FFE sequence^16^ (TE_1_ = 1.07 ms; TE_2_ = 1.9 ms; TR = 3.0 ms, FA = 10°, FOV = 250 × 371 × 200 mm^3^, AQR 1.8 × 1.8 × 3.6 mm^3^; SENSE factor = 2), using a 16 channel XL torso coil to receive the signal, was used to create a movie using the maximum intensity projections (MIP) of the water only images (Fig. 3). The movies allowed rotation of the colonic image giving 3D visualization and were useful in clarifying the position of the capsules at 24 h, if the T1 weighted TFE image was not conclusive.

**Figure 4:**
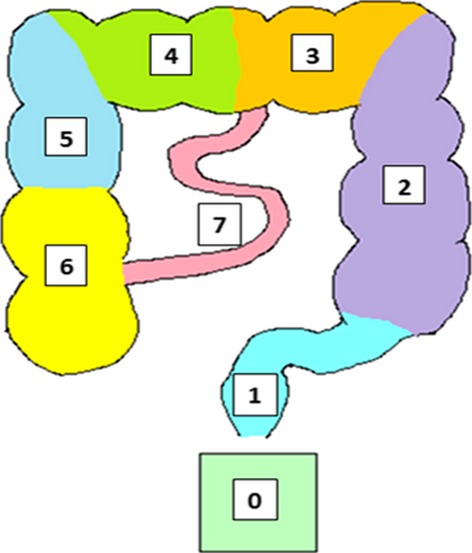
Segmented Colon. Showing the segmented colon used to score the MRI marker capsules at 24 h, where 0 = not found (presumed to be excreted), 1 = sigmoid and rectum, 2 = descending colon, 3 = left transverse colon, 4 = right transverse colon, 5 = upper ascending colon, 6 = lower ascending colon, 7 = small bowel.

From the MRI images a transit score was calculated by sub-dividing the bowel into eight sections (Fig. 4) and each capsule was scored according to its position in the colon at 24 h. On several data sets 1 or 2 capsules separated in position by several segments from the rest of the capsules (visualized together in a group). To reduce the effect of these outliers, due to the small number of pills used (five capsules compared to 20 ROM/day), a weighting factor was calculated for each capsule depending on the difference of the capsule score from the median capsule score. For a difference of 0 and 1 the weighting factor was 1, for all differences larger than 1 the weighting factor was the inverse of the difference. A weighted mean position score of the MRI marker capsules was thus determined for each volunteer. A non-weighted least square fit was applied to the MRI marker capsule scores and their corresponding ROM transit scores to calculate a transit time in hours for the MRI marker capsules. This is based on the equation: *y* = m*x* + c (Fig. 6A). Where *x* is the average MRI marker position, m and c are unique coefficients determined from the validation study (m = 0.03, c = −0.12), and *y* is the transit time in hours.

## Standard ROM test

The validated protocol described by Metcalf^5^ was used in this study. Subjects swallowed 20 ROMs on three consecutive days (days 1, 2, and 3) and an abdominal X-ray was taken on the following morning (day 4) immediately after an MRI scan, used to locate the MRI marker capsules consumed the day before (day 3). The ROMs were made of silicone tubing, impregnated with 13.5% barium, with the dimensions of 2.42 × 5.09 × 1.6 mm (Altimex, Nottingham, UK). The WGT was calculated in the standard way by counting the number of ROMs remaining on day 4 and multiplying by 1.2 to give a WGT in hours.

## Statistical analysis and power of studies

Statistics: For all data statistical analysis was carried out using the software, Prism 5 (GraphPad Software Inc, San Diego, CA, USA). The distribution of data were tested using the D'Agostino and Pearson omnibus normality test. The first primary outcome for this study was the correlation between OCTT measurements using the two test types. The second primary outcome was the correlation between the WGT measurements using the two test types. Since the data were not normally distributed the Spearman's rank correlation coefficient test was used to assess correlations. The secondary outcome for the study was to assess the reproducibility of the different methods described and we used the intra-class correlation coefficient test (ICC) to assess this.

Power calculations: Using the data from Horikawa *et al*.^17^ giving a mean colonic transit for healthy volunteers of 35.7 ± 12.9 h (mean ± SD), we calculated that for 80% power to detect a 25% difference in transit between the two methods, generally accepted as approximately the minimal clinically important difference, we would need 19 subjects. We increased the numbers to 21 to allow for dropouts and technical problems.

## Results

All subjects completed both studies with no adverse events, with the exception of one subject who did not attend the X-ray appointments. The breath test data from 1 volunteer, on visit 1, was omitted due to a high level of noise within the data produced.

### Comparison of the OCTT measured using the LUBT and MRI

**Figure 5:**
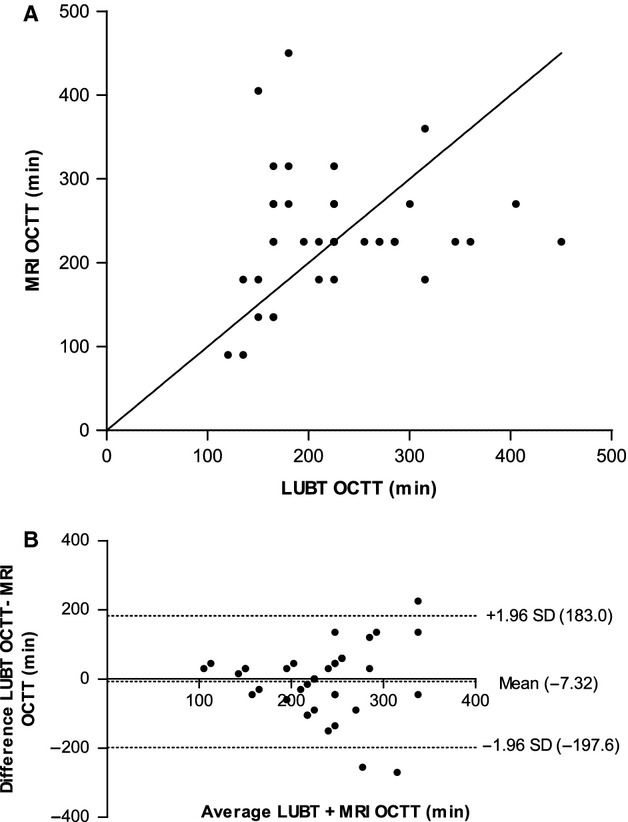
(A) Correlation between Lactose Ureide Breath Test (LUBT) and magnetic resonance imaging (MRI). Scatter plot with line of identity, comparing the orocecal transit time (OCTT) measured using the LUBT and MRI. The degree of correlation was assessed using the Spearman's rank correlation coefficient test, and we report a Spearman's *r*-value of 0.28 (not significant). (B) Agreement between OCTT measurements. Bland-Altman plot showing the average OCTT measured using the LUBT and MRI on the *x*-axis, and the difference between the OCTT measured using the two methods on the *y*-axis. This plot shows that there was a mean difference of −7.32 min between the LUBT and MRI (middle dotted line), with the limits of agreement ranging from 183.0 to −197.6 min (upper and lower dotted lines).

The median OCTT measured using the breath test was 225 min (IQR 165–278) and the median OCTT measured using the MRI based technique was also 225 min (IQR 180–270). Fig. 5A shows that there was weak correlation between OCTT measurements taken using the two different methods (*r*_s_ = 0.28, not significant). The Bland Altman plot in Fig. 5B shows the limits of agreement between the two methods. This graph shows that although there is a small mean difference of −7.32 min between measurements, the difference between measurements ranges from −197.6 min to 183.0 min, as indicated by the upper and lower dotted lines in Fig. 5B. There appears to be a tendency for the difference between measurements to increase the longer the transit time.

We used the ICC statistical test to assess the repeatability of the two tests on two separate occasions. The ICC for repeat OCTT measurement using the LUBT and MRI was 0.35 (*p* = 0.058) and 0.45 (*p* = 0.017) respectively. We also examined the inter-observer agreement between OCTT measurement using our new MRI based method, which gave an ICC of 0.44 (*p* = 0.002). Inter-observer agreement was not measured for the LUBT OCTT results as measurements were generated automatically using an in house analysis program.

### Comparison of the WGT measured using ROM and MRI markers capsules

**Figure 6:**
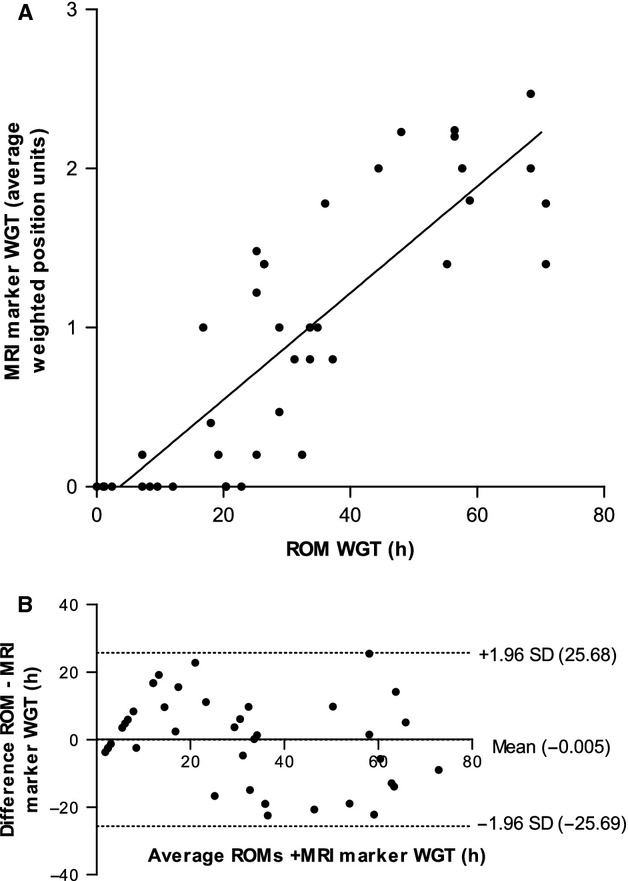
(A) Correlation between radio-opaque markers (ROM) and magnetic resonance imaging (MRI) markers. Plot comparing the whole gut transit time (WGT) measured using ROMs and MRI marker capsules showing the line of best fit as *y* = 0.03*x* ± 0.12. The degree of correlation was assessed using the Spearman's rank correlation coefficient test, and we report a Spearman's *r*-value of 0.85 (*p* < 0.0001). (B) Agreement between WGT measurements. Bland-Altman plot showing the average WGT measured using the ROMS and MRI marker capsules on the *x*-axis, and the difference between the WGT measured using the two methods on the *y*-axis. This plot shows that there was a mean difference of −0.005 h between test types (middle dotted line), with limits of agreement ranging from 25.68 to −25.69 h (upper and lower dotted lines).

The mean WGT measured using ROMs was 31 ± 3 h (SEM), whilst the median average weighted position score of the MRI marker capsules was 0.8 (IQR 0–1.6). We used the regression equation linking the two techniques to convert the average weighted capsule scores at 24 h to a WGT in hours, giving a median WGT of 28 h (IQR 4–50 h). Fig. 6A shows the correlation between the two methods is reasonably good (*r*_s_ = 0.85, *p* ≤ 0.0001). The Bland Altman plot in Fig. 6B shows the mean difference between WGT measurements was −0.005 h, with a range from −25.69 to 25.68 h as indicated by the upper and lower dotted lines on the plot in Fig. 6B.

The ICC for WGT values obtained on two separate study days using the ROMs and MRI marker capsule methods was also reasonable at 0.69 (*p* ≤ 0.001) and 0.61 (*p* = 0.001) respectively. We also examined the inter-observer agreement between WGT measurements using the ROM and MRI marker method, giving an ICC value of 0.995 (*p* ≤ 0.001) and 0.78 (*p* ≤ 0.001) for the two tests respectively.

In addition to measuring transit times, we used the 2D FFE MRI images obtained in study 1 to measure volunteers' colonic volumes (regional and total), both before and after a meal. In addition, we used single shot TSE images to assess SBWC as previously described and validated.^12^ In summary, we found no significant correlations between colonic volume, SBWC, and transit time (both OCTT and WGT; Table 1). In addition, we found no significant correlation between transit time (both OCTT and WGT) and volunteer demographics such as gender, BMI, anxiety, depression although there was a weak correlation between age and OCTT (Table 1). Although most studies find females to have slightly slower transit there is huge overlap and in our study males and females had very similar transit times median (IQR) 27.6 (3.7–45.4) and 25.6 (3.7–58.8) hours, respectively, *p* = 0.7.

**Table 1:** Correlations between MRI parameters and patient demographics

	OCTT (min)*	WGT (h)†
Age	*r*_s_ = 0.36 *p* = 0.02	*r*_s_ = −0.08 *p* = 0.61
Height (m)	*r*_s_ = −0.04 *p* = 0.81	*r*_s_ = −0.11 *p* = 0.50
Weight (kg)	*r*_s_ = 0.09 *p* = 0.56	*r*_s_ = −0.21 *p* = 0.18
BMI (kg/m^2^)	*r*_s_ = 0.13 *p* = 0.41	*r*_s_ = −0.23 *p* = 0.14
Anxiety score	*r*_s_ = −0.29 *p* = 0.08	*r*_s_ = 0.13 *p* = 0.42
Depression score	*r*_s_ = −0.15 *p* = 0.38	*r*_s_ = 0.16 *p* = 0.31
Total colonic volume at t360 (mL)	*r*_s_ = 0.26 *p* = 0.10	*r*_s_ = 0.16 *p* = 0.33
Ascending colon volume at t360 (mL)	*r*_s_ = 0.27 *p* = 0.08	*r*_s_ = 0.03 *p* = 0.86
Transverse colon volume at t360 (mL)	*r*_s_ = 0.02 *p* = 0.89	*r*_s_ = 0.13 *p* = 0.42
Descending colon volume at t360 (mL)	*r*_s_ = 0.29 *p* = 0.07	*r*_s_ = 0.17 *p* = 0.27
∆ t405-t360 Total colon volume (mL)	*r*_s_ = −0.07 *p* = 0.66	*r*_s_ = 0.02 *p* = 0.91
∆ t405-t360 Ascending colon volume (mL)	*r*_s_ = −0.19 *p* = 0.23	*r*_s_ = −0.10 *p* = 0.90
∆ t405-t360 Transverse colon volume (mL)	*r*_s_ = −0.11 *p* = 0.50	*r*_s_ = −0.16 *p* = 0.31
∆ t405-t360 Descending colon volume (mL)	*r*_s_ = −0.04 *p* = 0.81	*r*_s_ = −0.13 *p* = 0.43
Fasted SBWC (mL)	*r*_s_ = 0.17 *p* = 0.28	*r*_s_ = −0.08 *p* = 0.61
AUC SBWC (mL/min)	*r*_s_ = −0.01 *p* = 0.97	*r*_s_ = 0.08 *p* = 0.65

*r*_s_=Spearman's rank correlation coefficient.

^*^ Orocecal transit time (OCTT) measured using the Lactose Ureide breath test (LUBT).

^†^ Whole gut transit time (WGT) measured using MRI marker capsules.

## Discussion

When analyzing the data we found no difficulty in identifying the capsules in the MRI images and using the 3D rotating MIP movie the exact position of each capsule could be readily clarified if not conclusive from the T1 weighted TFE image. This is rather different from the ROM method when it can be difficult to identify the precise site of the ROMs located in the pelvic region on a plain X-ray.

We used a method to quantify the WGT similar to that used for tracer dispersion in scintigraphic images when quantifying colonic transit.^18^ However, the novel aspect of our MRI marker analysis is that the formula used to calculate the transit time takes into consideration the spread of the marker capsules position along the gut by looking at the difference of each capsule position from the median capsule position, using this to apply a weighting factor to each capsule score. This was done because it was noted that in the majority of healthy volunteers the transit marker capsules travelled along the gut as a group, but in some volunteers a few capsules separated substantially from the group, which heavily affects a simple mean position score. Although the use of the weighting factor for the MRI marker capsule scores made only a small change to the average median capsule position unit, 0.97 (non-weighted) vs 0.8 (weighted), it made a larger change to the longer transit times. Using the weighted score improved the Spearman's rank correlation coefficient with the ROM method from 0.7 (*p* < 0.0001) for non-weighted to 0.85 (*p* < 0.0001) for weighted.

A previous MRI feasibility study used five small eppendorf tubes filled with a Gd-DPTA/saline solution as markers of transit^19^ which gave an estimated colonic transit time of 41 ± 9 h in women and 31 ± 10 h in men, although their methodology and analysis were different from ours making direct comparison difficult. Our study builds on this previous study by designing a capsule which could be transferable to clinical practice, comparing the method against the widely used ROM method and using widely available MRI scans at a single 24 h time point instead of six time points over 60 h as in their method. The optimum assessment period (24 h *vs* 48 h) for measuring colonic transit has been evaluated for the scintigraphic method where a single dose is given and colonic transit assessed,^20^ which was a similar method to the one described here. This showed the lowest short-term intra-subject variation at 24 h. However, other studies suggested that 48 h does better especially for slower transits.^21^ It would be worthwhile and easy in future studies to include a 48 h scan to address this point.

We found a strong correlation between the WGT measured by MRI and the ROM method. Previous studies suggest that ROMs which are typically 2 mm diameter and the MRI marker capsules which are much larger (20 × 7 mm) may travel through the different regions of the gut at different rates. Previous work indicates that small pellets <2 mm diameter empty from the stomach during the digestive phase, whilst large capsules will empty more slowly after a meal, emptying during phase III of the MMC.^22^,^23^ This is supported by more recent studies comparing emptying of scintigraphic liquid phase markers given with a solid meal with a 25 × 12 mm wireless motility capsule.^24^ However, once in the small bowel and mixed with chyme, movement is unaffected by dosage form.^25^,^26^ Although it has been suggested that larger capsules will move ahead of smaller pellets in the colon,^27^ the similar WGT values seen with ROMs (29.7 h [IQR 22.4–45.7 h]) and the wireless motility capsules suggest that these minor differences do not significantly alter the final result^28^ and the values obtained for WGT were similar to those we observed. Previous animal studies suggested that when pills are fed with food, a pill density of 1.0 was associated with fastest transit and pills with a density greater than or less than 1.0 emptied more slowly.^29^ Human studies showed that, when taken fasting, pills with an extremely high density (2.4 g/cm^3^) empty slower than those with a density of 1.5 g/cm^3^ but lower densities were not evaluated.^30^,^31^ However, densities less that 1.0 would be expected to float as we have shown for lipids,^32^ and hence have delayed emptying.

Our imaging technique allows us to easily assess the position of the markers and assign accurately to upper or lower half of the ascending, transverse, and descending colon which increases accuracy compared with less fine grained assessment. However, the sigmoid is much more convoluted, divided into small segments and hence it is difficult to assign markers with such precision so we have grouped the sigmoid and rectum together.

As others have reported^33^ we found that reproducibility for WGT was better than for OCTT with an ICC value of 0.69 (*p* < 0.001) for the ROM method and 0.61(*p* = 0.001) for the MRI marker capsule method.

We were also interested to see if we could develop a purely MRI based method to quantify both WGT and OCTT so we also assessed the OCTT using MRI and compared it with the LUBT. The median OCTT value we measured using the LUBT was 225 min (IQR 165–278 min), which is slightly faster compared with the value of 292 ± 58 min quoted in a study where the LUBT was validated against scintigraphy but the meal used which consisted of one scrambled egg and two slices of bread was smaller than ours which may account for the differences.^14^ We found ICC values for repeat measurements of LUBT and MRI were both low at 0.35 (*p* = 0.058) and 0.46 (*p* = 0.017), suggesting OCTT depends on variables which were not easily controlled as others have reported.^21^ Since the extent of individual variability is similar for both techniques this suggests the variability reflects intrinsic biological variability rather than methodological variability.^33^

There have been many attempts to develop measurements of OCTT. The lactulose breath hydrogen test has been widely used in the past^34^ but it is known that the osmotic effect of the unabsorbable lactulose accelerates transit when compared with scintigraphy making it unsuitable as an assessment. Furthermore, interpretation of breath hydrogen is complex in precisely the conditions when small bowel transit is likely to be abnormal, since bacterial overgrowth is common and would give a spuriously short OCTT value. Unfortunately this is also likely to be true for the LUBT which, however, does not alter transit in the way that lactulose does, as the dose used is so small. The Wireless Motility Capsule (SmartPill® GI Monitoring System, the SmartPill Corporation, Buffalo, NY, USA)^35^ can also be used to assess OCTT measured from the time the pH rises on entering the duodenum to the time it falls on entering the colon. A median OCTT of 276 min was reported in a study using the standard eggbeater meal (194 Kcal).^35^ Its use is limited by cost but also in conditions of small bowel disease associated with narrowing (Crohn's disease, radiation ileitis etc.) when there is a risk of the pill getting stuck in the narrowing. A recent review concludes that scintigraphy is probably the best assessment of OCTT in clinical practice but warns that the technique is not standardized and the normal range is wide, a severe limitation of this test.^3^

Our MRI based technique for measuring the OCTT has been described previously and involves looking for the arrival of the high intensity head of a 362 Kcal rice pudding meal in the cecum.^11^ We obtained a median OCTT value of 225 min (IQR 180–270 min) for the same test meal using this new approach, which is in very close agreement with values reported previously using this method.^11^ Although we report the same median OCTT values using LUBT and MRI, with similar ranges, our results showed poor agreement between the two methods (Fig. 5B). One limitation to our MRI technique compared with the LUBT is that due to cost we were limited to scanning every 45 min, while we sampled every 10–15 min for the breath test. Another important consideration was that on occasions it was difficult to interpret the arrival of the head of the meal on the MRI images, particularly in cases where bright residues appeared in the cecum before or soon after eating the rice pudding meal which could cause confusion with the later actual arrival of the head of the meal. We compared two observers and showed weak agreement between measurements with an ICC of 0.44 (*p* = 0.002) suggesting that this would not be a useful measure as it depended too much on observer characteristics.

By contrast the MRI marker capsule method described is simple, involving just one visit for a set of MRI scans which takes round 5 min to perform and which are easily interpreted due to the detailed anatomical information provided. The MRI scans used are not specialist research scans but ones commonly available on most clinical MRI scanner platforms. We believe for these reasons that it could be widely adopted. Using our algorithm the results of the test given in hours would be very simple to interpret by the clinician and we observed strong inter-observer agreement between measurements using this methods, with an ICC of 0.78 (*p* < 0.001). Although in this study we used a 3 T MRI scanner to visualize the capsules at 24 h, we have recently shown that that method can also be used with 1.5 T MRI scanners (RC Spiller, unpublished data), and performed concurrently with the MRI OCTT method described, making the combined test easily transferable to hospital use. Moreover, as the T1 weighted 3D TFE sequence does not require the use of a dedicated torso coil to receive the RF signal, this further increases the portability and simplicity of the method for use in clinical practice. A dedicated torso coil can be used to acquire additional images which can be converted into rotating movies and these provide good spatial resolution which can be useful for further confirmation of the exact position of a capsule. The sequences used are available on all current MRI scanners and scanning time is very short at around 5 min. Since the images are easy to interpret a trained physicist could and does report the scans. The cost of MRI scanning is falling and if the test avoids the use of other investigations might well be cost effective. Our method has the advantage that we can simultaneously measure colonic transit and SBWC which will be useful when extending it to use in patients with various GI disorders of gastrointestinal function. It is non-invasive, highly patient acceptable and does not expose the patient to ionizing radiation allowing its use in young women and children, a group in whom constipation is common and assessment of transit often clinically useful.

## Abbreviations

AQR: acquired resolution
AXR: abdominal X-ray
DOTA: 1,4,7,10-tetraazacyclododecane-1,4,7,10-tetraacetic acid
FA: flip angle
FFE: fast field echo
FGIDs: functional gastrointestinal disorders
FOV: field of view
ICC: intraclass correlation coefficient
IQR: inter-quartile range
LUBT: Lactose Ureide breath test
MIP: maximum intensity projection
MMC: migrating motor complex
MRI: magnetic resonance imaging
OCTT: orocecal transit time
ROMs: radio-opaque markers
SBWC: small bowel water content
SEM: standard error of the mean
TE: echo time
TR: repetition time
TSE: turbo spin echo
WGT: whole gut transit time

## Supporting Information

**Video S1.** Representative example of rotating MRI movie showing five MRI marker capsules in the colon of a healthy volunteer 24 h after ingestion.

## References

[1] Jones J, Boorman J, Cann P (2000). British Society of Gastroenterology guidelines for the management of the irritable bowel syndrome. Gut.

[2] Drossman DA (1999). The functional gastrointestinal disorders and the Rome II process. Gut.

[3] Rao SS, Camilleri M, Hasler WL (2011). Evaluation of gastrointestinal transit in clinical practice: position paper of the American and European Neurogastroenterology and Motility Societies. Neurogastroenterol Motil.

[4] Chang L, Drossman DA (2010). Rome Foundation endpoints and outcomes conference 2009: optimising clinical trials in FGID. J Gastroenterol.

[5] Metcalf AM, Phillips SF, Zinsmeister AR, MacCarty RL, Beart RW, Wolff BG (1987). Simplified assessment of segmental colonic transit. Gastroenterology.

[6] Camilleri M, Colemont LJ, Phillips SF (1989). Human gastric-emptying and colonic filling of solids characterized by a new method. Am J Physiol.

[7] Weitschies W, Blume H, Monnikes H (2010). Magnetic marker monitoring: high resolution real-time tracking of oral solid dosage forms in the gastrointestinal tract. Eur J Pharm Biopharm.

[8] Camilleri M, Thorne NK, Ringel Y (2010). Wireless pH-motility capsule for colonic transit: prospective comparison with radiopaque markers in chronic constipation. Neurogastroenterol Motil.

[9] Hahn T, Kozerke S, Schwizer W, Fried M, Boesiger P, Steingoetter A (2011). Visualization and quantification of intestinal transit and motor function by real-time tracking of F-19 labeled capsules in humans. Magnet Reson Med.

[10] Szarka LA, Camilleri M (2012). Methods for the assessment of small-bowel and colonic transit. Semin Nucl Med.

[11] Marciani L, Cox EF, Hoad CL (2010). Postprandial changes in small bowel water content in healthy subjects and patients with irritable bowel syndrome. Gastroenterology.

[12] Hoad CL, Marciani L, Foley S (2007). Non-invasive quantification of small bowel water content by MRI: a validation study. Phys Med Biol.

[13] Vassallo M, Camilleri M, Phillips SF, Brown ML, Chapman NJ, Thomforde GM (1992). Transit through the proximal colon influences stool weight in the irritable-bowel-syndrome. Gastroenterology.

[14] Geypens B, Bennink R, Peeters M (1999). Validation of the lactose-[C-13]ureide breath test for determination of orocecal transit time by scintigraphy. J Nucl Med.

[15] Placidi E (2011). Magnetic Resonance Imaging of Colonic Function.

[16] Eggers H, Brendel B, Duijndam A, Herigault G (2011). Dual-echo Dixon imaging with flexible choice of echo times. Magn Reson Imaging.

[17] Horikawa Y, Mieno H, Inoue M, Kajiyama G (1999). Gastrointestinal motility in patients with irritable bowel syndrome studied by using radiopaque markers. Scand J Gastroenterol.

[18] Krevsky B, Malmud LS, Dercole F, Maurer AH, Fisher RS (1986). Colonic transit scintigraphy - a physiological approach to the quantitative measurement of colonic transit in humans. Gastroenterology.

[19] Buhmann S, Kirchhoff C, Ladurner R, Mussack T, Reiser MF, Lienemann A (2007). Assessment of colonic transit time using MRI: a feasibility study. Eur Radiol.

[20] Deiteren A, Camilleri M, Bharucha AE (2010). Performance characteristics of scintigraphic colon transit measurement in health and irritable bowel syndrome and relationship to bowel functions. Neurogastroenterol Motil.

[21] Cremonini F, Mullan BP, Camilleri M, Burton DD, Rank MR (2002). Performance characteristics of scintigraphic transit measurements for studies of experimental therapies. Aliment Pharmacol Ther.

[22] Davis SS, Hardy JG, Fara JW (1986). Transit of pharmaceutical dosage forms through the small intenstine. Gut.

[23] Proano M, Camilleri M, Phillips SF, Brown ML, Thomforde GM (1990). Transit of solids through the human colon - regional quantification in the unprepared bowel. Am J Physiol.

[24] Maqbool S, Parkman HP, Friedenberg FK (2009). Wireless capsule motility: comparison of the SmartPill(A (R)) GI monitoring system with scintigraphy for measuring whole gut transit. Digest Dis Sci.

[25] Adkin DA, Davis SS, Sparrow RA, Wilding IR (1993). Colonic transit of different sized tablets in healthy-subjects. J Control Release.

[26] Malagelada JR, Robertson JS, Brown ML (1984). Intestinal transit of solid and liquid components of a meal in health. Gastroenterology.

[27] Hardy JG, Wilson CG, Wood E (1985). Drug delivery to the proximal colon. J Pharm Pharmacol.

[28] Rao SS, Kuo B, McCallum RW (2009). Investigation of colonic and whole-gut transit with wireless motility capsule and radiopaque markers in constipation. Clin Gastroenterol Hepatol.

[29] Meyer J, Dressman J, Fink A, Amidon G (1985). Effect of size and density on canine gastric emptying of nondigestible solids. Gastroenterology.

[30] Devereux JE, Newton JM, Short MB (1990). The influence of density on the gastrointestinal transit of pellets. J Pharm Pharmacol.

[31] Clarke GM, Newton JM, Short MD (1993). Gastrointestinal transit of pellets of differing size and density. Int J Pharmaceut.

[32] Boulby P, Gowland P, Adams V, Spiller RC (1997). Use of echo planar imaging to demonstrate the effect of posture on the intragastric distribution and emptying of an oil/water meal. Neurogastroenterol Motil.

[33] Degen LP, Phillips SF (1996). Variability of gastrointestinal transit in healthy women and men. Gut.

[34] Miller MA, Parkman HP, Urbain JLC (1997). Comparison of scintigraphy and lactulose breath hydrogen test for assessment of orocecal transit - Lactulose accelerates small bowel transit. Digest Dis Sci.

[35] Sarosiek I, Selover KH, Katz LA (2010). The assessment of regional gut transit times in healthy controls and patients with gastroparesis using wireless motility technology. Aliment Pharm Therap.

