# Supplementary material for: Novel MRI tests of orocecal transit time and whole gut transit time: studies in normal subjects
Source: Neurogastroenterol Motil. 2013 Oct 25;26(2):205–14. doi: 10.1111/nmo.12249 (PMC4285997; doi:10.1111/nmo.12249)
Supplement: Supplementary file 2 [file nmo0026-0205-sd2.doc]

**Supplemental MRI movie legend.**

Representative example of rotating MRI movie showing 5 MRI marker capsules in the colon of a healthy volunteer 24 hours after ingestion
